# Minocycline Synergizes with N-Acetylcysteine and Improves Cognition and Memory Following Traumatic Brain Injury in Rats

**DOI:** 10.1371/journal.pone.0012490

**Published:** 2010-08-31

**Authors:** Samah G. Abdel Baki, Ben Schwab, Margalit Haber, André A. Fenton, Peter J. Bergold

**Affiliations:** Departments of Physiology and Pharmacology, State University of New York-Downstate Medical Center, Brooklyn, New York, United States of America; Virginia Commonwealth University, United States of America

## Abstract

**Background:**

There are no drugs presently available to treat traumatic brain injury (TBI). A variety of single drugs have failed clinical trials suggesting a role for drug combinations. Drug combinations acting synergistically often provide the greatest combination of potency and safety. The drugs examined (minocycline (MINO), N-acetylcysteine (NAC), simvastatin, cyclosporine A, and progesterone) had FDA-approval for uses other than TBI and limited brain injury in experimental TBI models.

**Methodology/Principal Findings:**

Drugs were dosed one hour after injury using the controlled cortical impact (CCI) TBI model in adult rats. One week later, drugs were tested for efficacy and drug combinations tested for synergy on a hierarchy of behavioral tests that included active place avoidance testing. As monotherapy, only MINO improved acquisition of the massed version of active place avoidance that required memory lasting less than two hours. MINO-treated animals, however, were impaired during the spaced version of the same avoidance task that required 24-hour memory retention. Co-administration of NAC with MINO synergistically improved spaced learning. Examination of brain histology 2 weeks after injury suggested that MINO plus NAC preserved white, but not grey matter, since lesion volume was unaffected, yet myelin loss was attenuated. When dosed 3 hours before injury, MINO plus NAC as single drugs had no effect on interleukin-1 formation; together they synergistically lowered interleukin-1 levels. This effect on interleukin-1 was not observed when the drugs were dosed one hour after injury.

**Conclusions/Significance:**

These observations suggest a potentially valuable role for MINO plus NAC to treat TBI.

## Introduction

Despite its high prevalence in the population, only palliative treatments are available for TBI [1], [2]. A recent NIH workshop on combination drug therapy for TBI concluded that past failures in clinical trials resulted, in part, from preclinical testing that selected drugs with insufficient potency [2]. Combination therapy has become the standard of care for many common diseases including cancer, hypertension, tuberculosis and AIDS [3], [4], [5], [6]. Combination therapies were developed to treat these diseases since monotherapy was no longer effective. Combination therapy may also be advantageous for TBI even though monotherapy has not shown to be effective. TBI damages the brain in many ways; multiple drugs with disparate mechanisms of action may better interfere with these injury mechanisms. Greater potency can be achieved using synergistic drug combinations, but drug pairs may have synergistic adverse effects as well [7]. Adverse effects in drug combinations can potentially be avoided by combining FDA-approved drugs with known drug interactions.

Inadequate preclinical testing may have also contributed to the failure of clinical trials [1], [2]. Preclinical testing has evaluated lesion volume; cell loss; blood-brain barrier permeability; intracranial pressure; brain edema; and measures of apoptosis, inflammation, or oxidative stress as markers of drug efficacy [1], [2]. These markers measure important components of brain injury, yet drugs that improved these outcomes failed clinical trials [2].

A hierarchy of behavioral tasks has been developed that places a high cognitive demand on rats [8]. This hierarchy may provide a more stringent test for drug efficacy than previous behavioral or histological tests [9]. The first two tasks of the behavioral hierarchy were the open field and passive place avoidance [8]. These tests examined basic motor, sensory and motivational parameters. Rats receiving moderate or sham-CCI have been previously shown to perform similarly on the open field and passive place avoidance tests [8]. The equivalent performance sham- or moderate-CCI injured rats in the open field and passive place avoidance suggests intact innate behaviors, as well as similar levels of anxiety, motivation, sensory and motor ability [8]. Active place avoidance, the third and final task in the hierarchy tested whether rats with moderate- or sham-CCI could learn to avoid a stationary shock zone on a rotating arena. Optimal performance of active place avoidance requires perceptual segregation since rats must attend to distal, stationary visual cues to identify the location of the shock zone while ignoring irrelevant proximal, rotating olfactory cues that are not informative [10], [11]. The active place avoidance task places a high cognitive demand on rats that goes beyond memory, although the task is also sensitive to short-term and long-term memory impairments [11], [12]. Injecting one hippocampus with the sodium channel blocker tetrodotoxin completely impaired active place avoidance learning [13]. The identical tetrodotoxin treatment did not prevent rats from learning the location of the escape platform in the Morris water maze, another test of spatial memory and navigation [9]. Sham-injured rats optimally learned the massed version of the active place avoidance task that consisted of six 10-minute trials with a 10-minute intertrial interval [8]. In contrast, moderate CCI produced large, long-lasting active place avoidance deficits during massed training [8]. Cognitive deficits were responsible for the impaired active place avoidance since injured rats showed no motivational, behavioral, sensory or motor deficits. This study used the active place avoidance task to examine whether drugs can limit cognitive deficits produced by moderate CCI. Rats receiving mild CCI or moderate CCI showed similar performance on the first two tasks of the hierarchy. On the third task, active place avoidance, rats receiving moderate CCI showed large and permanent deficits; in contrast, no deficits were seen in rats receiving mild CCI [8].

TBI damages both white and grey matter, but markers of white matter injury are now thought to better correlate with higher brain function [14]. TBI readily injures long white matter tracts such as the corpus callosum, fimbria-fornix, hippocampal commissure and internal capsule [15]. This study assayed myelin loss in these white matter regions after moderate CCI.

Clinical or experimental TBI produces a large and rapid increase in interleukin-1 beta (IL-1) [16], [17], [18]. Il-1 levels reach a maximum in the hippocampus 1–2 hours after injury [18]. Perfusion into the brain of the interleukin-1 receptor antagonist, reduced lesion volume and improved learning of platform location in the Morris water maze [19]. Conversely, IL-1β infusion increased lesion volume in the fluid percussion model of TBI [20]. These observations suggest a key role for IL-1β in the pathophysiology of TBI.

The present study examined the effects of both drugs and drug combinations on active place avoidance learning during massed training as well as during spaced training, consisting of a single 20-minute trial per day for 15 days. Massed training tests for learning mediated by short-term task retention plus perceptual segregation; spaced active avoidance also tests for 24-hour retention [11], [12]. This study identified MINO plus NAC as a synergistic drug combination based on their ability to improve performance on the active place avoidance task. The effects of MINO plus NAC, individually and in combination, were also examined on prevention of myelin loss and IL-1β production.

## Results

### MINO improved active place avoidance following CCI

**Figure 1:**
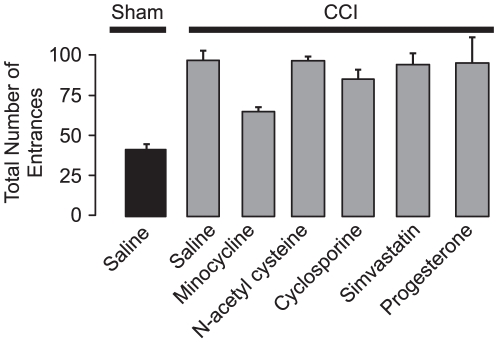
MINO provided a modest improvement of active place avoidance following moderate CCI. The total number of entrances into the shock zone was assayed in the 6 trials of active place avoidance training.

Five drugs - MINO, NAC, cyclosporine, simvastatin and progesterone - were selected because they were previously reported to limit TBI in rats [1], [18], [21], [22], [23], [24], [25]. Seven groups were tested: (1) Sham-CCI-saline; (2) CCI-saline; (3) CCI-MINO; (4) CCI-NAC; (5) CCI-cyclosporine; (6) CCI-simvastatin; (7) CCI-progesterone. Seven days following surgery, rats were assayed in the open field and passive place avoidance tasks. No differences were observed suggesting that all groups had similar innate, sensory and motor function (Table 1). The following day all groups were tested on massed active place avoidance (Figure 1). The total number of entrances in the massed training trials of active place avoidance showed a significant group effect of drug treatment with sham-CCI-saline rats having fewer entrances than the CCI-MINO group (F_6,35_ = 50.83; p<0.0001, post-hoc: p's<0.001) (Figure 1). All groups showed a significant trial effect demonstrating that some groups learned while others did not (F_5,175_ = 280.22, p<0.0001). To further analyze whether individual groups improved across trials, one-way ANOVA showed no significant improvement in the CCI-cyclosporine, CCI-simvastatin or CCI-progesterone and CCI-saline groups. Although there were differences among in task acquisition, all groups were able to reduce the number of entrances (F_4,25_ = 146.43, p<0.01). These differences were further supported by a significant trial effect (F_5,125_ = 285.31, p<0.01) suggesting that learning across trials also differed among groups. No interaction was noted between the factors of group and trials (F_20,125_ = 9.24, p = 0.41) indicating that changes in group and trials were independent. The CCI-cyclosporine, CCI-simvastatin or CCI-progesterone and CCI-saline group showed no significant improvement across trials (1- way ANOVA; CCI-cyclosporine, F_5,25_ = 28.6, p = 0.33; CCI-simvastatin, F_5,25_ = 33.65, p = 0.25; CCI-progesterone, F_5,25_ = 36.28, p = 0.31; CCI-saline, F_5,25_ = 44.61, p = 0.28). Only MINO improved active place avoidance learning during massed training (1 way ANOVA, F = 54.26, p<0.01) even though all the drugs were administered at doses that were effective at reducing TBI [1], [18], [21], [22], [23], [24], [25]. MINO was therefore chosen as one arm of the combination therapy to treat CCI.

**Table 1:** Measurements of rat behavior during open field, passive place avoidance and active place avoidance.

Task	Parameter	Sham CCI- Saline	CCI-saline	CCI- CYCLO	CCI-SIM	CCI-PROG	CCI-MINO	CCI-NAC	CCI-MINO plus NAC
Open Field	Total Distance	26.8±0.98	25.4±1.5	27.1±3.7	24.2±3.5	26.3±2.7	27.7±2.2	28.0±1.7	26.3±1.7
Passive PlaceAvoidance	Shock zone entrances	13±0.75	18±0.89	18±0.77	22±0.76	15±0.96	14±0.49	16±0.42	14±0.42
	Total Distance	7.8±0.3	7.3±0.5	6.9±0.5	7.7±0.7	7.1±0.7	6.4±0.6	6.7±0.5	7.0±0.6
Active PlaceAvoidance	Speed	5.8±0.3	5.1±0.4	5.7±0.5	4.3±0.4	6.1±0.3	5.6±0.2	5.0±0.3	5.4±0.3
	Linearity	0.60±0.03	0.64±0.04	0.71±0.04	0.75±0.05	0.66±0.01	0.67±0.03	0.65±0.03	0.62±0.03
	Shocks/Entrance	0.4±0.2	1.2±0.02	1.3±0.1	1.2±0.1	1.1±0.04	1.0±0.04	1.1±0.04	1.1±0.04
	Time to first entrance	472.5±63.4**	46.5±5.6	39.8±5.1	42.9±3.8	45.6±2.8	146.0±33.4*	52.9±7.3	243±24.0**

Rats received either sham- or moderate-CCI. Beginning one hour after surgery the rats received either saline, cyclosporine (CYCLO) simvastatin (SIM), progesterone (PROG), minocycline (MINO), n-acetyl cysteine (NAC) or MINO plus NAC. Seven days later all groups were tested on the hierarchy of three behavioral tasks. In the open field test, there was no effect of treatment on total distance traveled (F_7,40_ = 0.40, p>0.8). In passive place avoidance, there was no effect of treatment on either average distance over 4 trials or shock zone entrances (total distance, F_7,40_ = 0.67, p>0.6; entrances, F_7,40_ = 0.48, p>0.8). In massed active place avoidance testing on the 6^th^ trial of the first day, there was no effect of treatment on speed (F_7,40_ = 0.44, p>0.8) or linearity (F_7,40_ = 0.25, p>0.9)). The number of shocks per entrance in CCI-saline treated rats was not changed by the drugs individually or in combination (F_7,40_ = 0.39, p>0.6). In contrast, there was a significant treatment effect with MINO or MINO plus NAC significantly improved time to first entrance (F_4,35_ = 26.7, p<0.0001; **p<0.001, *p<0.05; post-hoc test).

### MINO plus NAC synergistically improved active place avoidance following CCI

**Figure 2:**
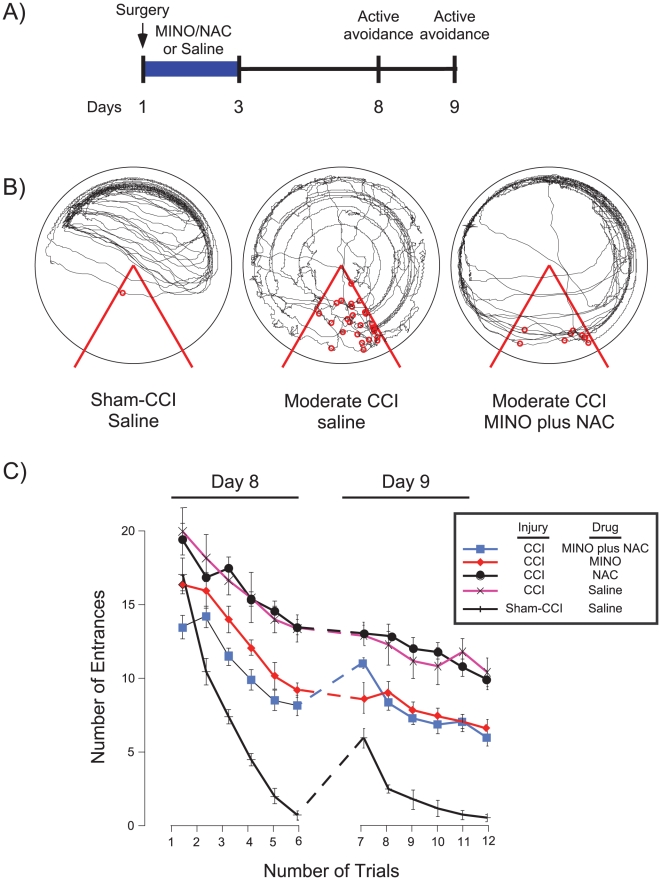
MINO plus NAC synergistically improved active place avoidance after massed training. Panel A, Rats received either CCI or sham-CCI. One hour, one or days later the sham-injured the CCI-injured rats were divided into 4 groups; one group received saline treatment. The remaining CCI-injured rats received either MINO or NAC alone, or the combination of MINO plus NAC. Saline or drug treatments were administered 1 hour, 1 day and 2 days after injury. Rats received active place avoidance training 8 and 9 days after injury. On the 8th and 9th day following CCI, the number of shock zone entrances was measured. **Panel B**, Representative tracks of rats in the sham-CCI-saline, CCI-saline or CCI-MINO plus NAC groups on the 6^th^ trial on the first day of active avoidance training. Red lines indicate the boundaries of the shock zone and the red circles indicate the location were a rat received a shock. **Panel C**, Summary of the number of shock zone entrances over the two days of active place avoidance.

MINO was first combined with cyclosporine. Within 4 days after injury, all rats died after receiving MINO, cyclosporine (n = 4). This contrasts with the 100% viability of rats that received MINO or cyclosporine as single drugs. NAC was selected as the next drug to be tested given its extensive safety record at the dose tested [26]. Rats were divided into five groups to test whether NAC had an effect on the ability of MINO to improve active place avoidance; (1) Sham-CCI-saline; (2) CCI-saline; (3) CCI-MINO; (4) CCI-NAC; (5) CCI-MINO plus NAC. At one week following injury no difference in open field and passive avoidance was observed, suggesting similar abilities to initiate and inhibit exploration, as well as sense and avoid shock (Table 1). All groups then received two days of 6 trials of active avoidance (Figure 2A). Group differences were observed in both the representative paths during trial 6 and the learning curves during massed active place avoidance training (Figures 2B and C). The sham-injured group receiving saline learned the task rapidly, entering the shock zone only once on trial 6. The injured groups receiving MINO or MINO plus NAC were partially impaired as their learning curves were initially flat; improvement became evident after trial 3, with a less steep learning curve than the sham-CCI-saline group. By trial 6, the CCI-MINO or CCI-MINO plus NAC groups had fewer than 8 shock zone entrances. The CCI-saline or CCI-NAC groups were more profoundly impaired. Their performance was worse than the other groups on trial 1, suggesting there was limited within-trial learning. By trial 6, they entered the shock zone more than 14 times, which was no better than the other groups on their first trial. The CCI-saline or CCI-NAC groups tended not to avoid the shock zone, instead they rapidly left the zone only after receiving a shock, suggesting that they lowered the number of entrances by rapidly running after being shocked. A comparison of the representative tracks in figure 2B provides evidence of this behavior. The sham-CCI track showed smooth trajectories as the rat actively avoided the shock zone. In contrast, the concentric circular paths of the track of CCI-saline group illustrates that the rat remained motionless as it was passively carried into the shock zone by the clockwise arena rotation. Therefore, the locations of the rat when it was shocked were largely located in the counter-clockwise half of the shock zone. In contrast, the MINO plus NAC-treated group, like the sham-CCI-saline group, only became active as they approached the shock zone. The tracks also show that MINO plus NAC-treated animals were less successful than the sham-CCI animals to avoid being shocked. Although there were group differences, all groups were able to reduce the number of entrances (F_4,25_ = 146.43, p<0.01). This confirmed group differences in task acquisition. These differences were further supported by a significant trial effect (F_5,125_ = 285.31, p<0.01), yet no interaction was noted between the factors of group and trials (F_20,125_ = 9.24, p = 0.41). Post-hoc tests further confirmed that the Sham-CCI-saline, CCI-MINO plus NAC, and CCI-MINO had reduced shock zone entrances as compared to the CCI-saline, and CCI-NAC groups which did not differ.

An important, surprising feature of learning curves was on the 1st trial of the second day (trial 7) of active place avoidance testing (Figure 2C). Comparing avoidance on trial 7 confirmed a significant group effect (F_1,14_ = 6.53; *p = 0.02). Even though the CCI-MINO and CCI-MINO plus NAC groups had similar avoidance throughout day 1, the CCI-MINO group had more entrances on trial 7. This observation suggested that the NAC synergistically improved 24-hour retention since the MINO plus NAC group performed better than MINO group despite no effect of NAC (post-hoc, p<0.01).

**Figure 3:**
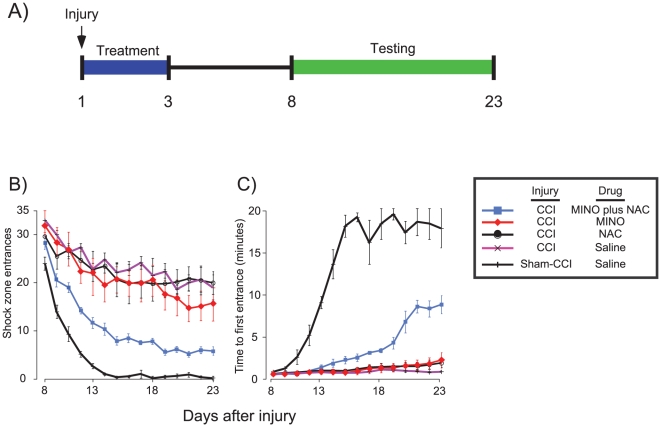
MINO plus NAC synergistically improved active place avoidance during spaced training that requires 24-hour memory. **Panel A**, Experimental Design. Rats received moderate CCI or sham-injury. Saline or drug treatments were administered 1 hour, 1 day and 2 days after injury. Beginning 8 days after injury, rats received spaced training of active place avoidance consisting of a 20-minute trial each day for 15 days. **Panel B**, The number of entrances into the shock zone measured overall avoidance learning. **Panel C**, Time to 1st entrance into the shock zone measured learning that depended on 24-hour retention. These data suggest that long-term memory was improved by MINO plus NAC but not MINO. MINO plus NAC acted synergistically since the improvement in time to enter the shock zone occurred only with co-administration of both drugs.

To confirm and further elucidate a potential synergy between MINO plus NAC, a second study was performed using spaced active place avoidance training because improved avoidance in this protocol relies on both 24-hour retention across sessions as well as within-session processes (Figure 3A). An additional five groups of rats were evaluated (Sham CCI-saline; CCI-saline; CCI-MINO; CCI-NAC; and CCI-MINO plus NAC). All groups performed similarly in the open field and passive avoidance tasks (table 1). In the spaced version of active place avoidance, group differences were apparent in the learning curves (F_4,25_ = 34.68; p<0.01; Figures 3B and C). The effect of trial was also significant indicating that some groups learned to reduce the number of entrances across trials (F_14,350_ = 72.86; p<0.01). There was no interaction between the factors of group and trial (F_56,350_ = 3.83; p = 0.26). Time to first entrance showed a similar pattern (Figure 3C). The sham CCI-saline group steadily increased in the ability to delay entering the shock zone, which increased to over 18 minutes. The CCI-MINO plus NAC group showed a less steep increase that plateaued at more than 8 minutes. This indicated a substantial improvement of avoidance learning and long-term memory. Rats in the remaining groups did not increase the time to first entrance beyond one minute, confirming a severe impairment of long-term memory seen in the earlier, massed protocol of active place avoidance (Figure 2C).

By the 7^th^ trial of spaced active place avoidance training, the sham-CCI-saline group reached asymptote on the parameters of number of entrances and time to first entrance (Figures 3B and C). The other groups reached asymptote only in the number of entrances. Therefore, statistical comparisons were performed on the 7^th^ trial of all groups. There was a significant effect of group (1-way ANOVA, F_4,25_ = 37.26, p<0.01). The sham-CCI-saline rats optimally avoided the shock zone and post-hoc comparisons confirmed that the CCI-MINO plus NAC group had significantly fewer entrances than the CCI-MINO group. The CCI-MINO group, however, performed better than the groups receiving NAC or saline. Thus total entrances, a parameter that is affected by both within-session learning and memory between sessions, showed a synergistic interaction between MINO plus NAC since NAC enhanced MINO action yet was ineffective as a single drug.

The time to 1^st^ entrance, the parameter that most directly measures 24-hour memory also showed drug synergy. The effect of group was significant (F_4,25_ = 206.15, p<0.01) as was the effect of trial (F_14,50_ = 62.42, p<0.01), indicating learning in at least some of the groups. The interaction between group and trial was not significant (F_56,350_ = 30.72, p = 0.33). At the end of training, on day 15, there was a significant effect of groups (Figure 3C). Post-hoc tests confirmed that the CCI-MINO plus NAC group had an elevated time to 1^st^ entrance as compared to the CCI-saline, CCI-NAC, and CCI-MINO groups, which did not differ. The MINO plus NAC group did not increase time to 1^st^ entrance as well as the sham CCI-saline group. These data provide strong evidence for drug synergy since MINO plus NAC combination was highly effective while the drugs were ineffective as monotherapy.

### Prevention of myelin loss by MINO plus NAC following TBI

After the completion of spaced active place avoidance training, rats were sacrificed, coronal brain sections were prepared and the loss of cortical tissue at the impact site was assayed. There was no significant effect of treatment suggesting that MINO or NAC, individually or in combination, has little effect on lesion volume (expressed as the percent difference from contralateral hemisphere) CCI-saline, 100.0±4.9%; CCI-MINO, 92.2±7.8%; CCI-NAC, 86.0±6.1%; CCI-MINO plus NAC, 87.4±6.4%; F_3,36_ = 1.1, p>0.4).

**Figure 4:**
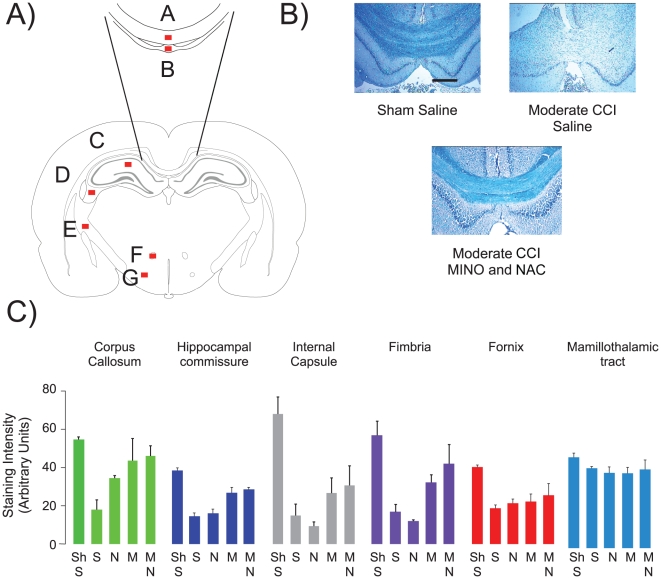
MINO plus NAC prevented myelin loss. **Panel A**, Schematic of the regions of interest (ROIs) from a coronal section located −3.36 mm from Bregma [40]. The ROIs were: corpus callosum (A), dorsal hippocampal commissure (B), stratum radiatum (C), fimbria (D), internal capsule (E), fornix (F), mammilothalamic tract (G). **Panel B**, Representative images of corpus callosum and dorsal hippocampal commissure stained with luxol fast blue. B1, Sham-CCI-saline; B2 CCI-saline; B3, CCI-MINO plus NAC. The scale bar corresponds to 100µm.

Myelin loss was assayed in adjacent coronal sections using the myelin-specific stain Luxol fast blue. Six white matter structures were examined- corpus callosum, dorsal hippocampal commissure, fimbria, internal capsule, fornix and mamallothalamic tract (Figure 4A). Moderate CCI reduced myelin content in many, but not all white matter regions and there were significant effects of group in all regions except the mamallothalamic tract suggesting impact of the drug treatments (corpus callosum, F_4,10_ = 8.85, p<0.01; hippocampal commissure, F_4,10_ = 25.75, p<0.0001; fimbria, F_4,10_ = 11.44, p<0.01; internal capsule, F_4,10_ = 13.15, p<0.01; fornix, F_4,10_ = 5.76, p<0.01; mamallothalamic tract, F_4,10_ = 1.20, p>0.3) (Figure 4B, C). NAC treatment limited myelin loss in the corpus callosum, but not in the other white matter tracts (post-hoc, p<0.05). MINO or MINO plus NAC treatment were similarly effective in preventing myelin loss (post-hoc, p = 0.23) (Figure 4C). These data suggest that MINO plus NAC prevented myelin loss after moderate CCI. Unlike the synergistic improvement in behavior, MINO plus NAC showed an indifferent drug effect since MINO plus NAC was no more effective than MINO alone.

### MINO plus NAC synergistically inhibit IL-1β production when dosed before moderate CCI

**Figure 5:**
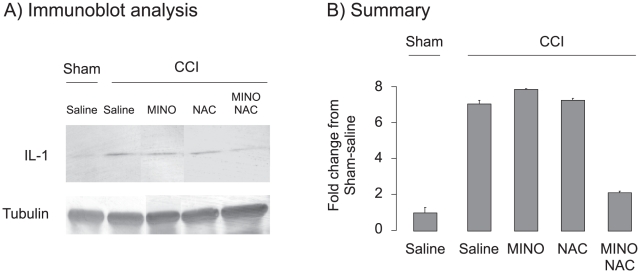
MINO or MINO plus NAC prevented IL-1β formation when administered before moderate CCI. Rats were dosed with saline, MINO, NAC or both drugs 3 hours prior to sham- or moderate CCI. The rats were sacrificed one hour after surgery; and IL-1β and tubulin levels were assayed from protein extracts prepared from hippocampus. **Panel A**, Representative Il-1β and tubulin immunoblot analysis, **Panel B**, Summary of immunoblot analysis.

Hippocampal IL-1β rapidly increases following TBI [17]. IL-1β levels were lowered by treatment with either MINO or NAC. [17], [18], [27], [28], [29]. To test whether the MINO, NAC drug combination lowered IL-1β levels, five groups (Sham-CCI-saline; CCI-saline; CCI-MINO; CCI-NAC; and CCI-MINO plus NAC) were examined. Rats were treated with saline or drugs three hours prior to sham-CCI or CCI, and hippocampal IL-1β levels were assayed one hour later (Figure 5). There was a significant effect of treatment (F_4,15_ = 14.84, p<0.01) with an increase in hippocampal IL-1β levels following moderate CCI that was significantly blocked by MINO plus NAC (post-hoc, p<0.001). Surprisingly, when dosed 3 hours prior to injury, neither MINO nor NAC as single drugs blocked the IL-1β increase despite their efficacy as a combination (post-hoc, p = 0.6). These data suggest that MINO plus NAC act synergistically to lower IL-1β levels when dosed before moderate CCI.

The dosing of drugs prior to injury provided insight into drug action but did not address whether the post-injury dosing of MINO plus NAC also regulated IL-1β levels after moderate CCI. To test whether these drugs acted similarly when dosed after injury, rats received either saline or MINO plus NAC one hour after moderate CCI. Four hours after injury, the rats were sacrificed and hippocampal IL-1β levels were measured. Western blot analysis showed no significant change in IL-1b levels in the hippocamppi of CCI-treated rats receiving post-injury treatments of either saline or MINO plus NAC (saline, 1.0±1.9 (n = 4); MINO plus NAC, 1.45±0.42 (n = 4); t_6_ = 0.24; p>0.05). These data suggest that MINO plus NAC did not affect hippocampal IL-1β levels when dosed one hour after injury.

## Discussion

This study identified three actions of the drug combination of MINO plus NAC following moderate CCI: (1) MINO plus NAC synergistically prevented cognitive deficits by improving learning as well as memory spanning at least 24-hours in the active place avoidance task (Figures 2 and 3), (2) myelin loss was prevented by either MINO alone or MINO plus NAC (Figure 4), and (3) MINO plus NAC synergistically prevented IL-1β formation when dosed before, but not after injury (Figure 5).

MINO plus NAC synergistically improved both task acquisition and reduced memory loss when treatment began one hour following moderate CCI (Figures 2 and 3). Moderate CCI was previously shown to produce large, long-lasting deficits in active place avoidance during massed training that prevented further testing if there was a further impairment of short- or long-term task retention [8]. MINO- and MINO plus NAC-treated animals acquired active place avoidance during massed training, which allowed us to examine if the drugs also improved short- and long-term task retention (Figure 2 and 3). MINO-treated animals had short-term retention of the massed active avoidance task (Figure 2). During the spaced protocol of active place avoidance, despite a modest decrease in the number of entrances, the MINO plus NAC-treated rats showed only a slight improvement of time to first entrance into the shock zone suggesting no long-term retention of shock zone location (Figure 3). Thus, the modest improvement in number of entrances is unlikely to be due to memory for the shock zone itself. Rather, it is likely due to non-specific, non-spatial learning or adoption of a sub-optimal behavioral strategy such as remaining motionless until receiving a shock. The MINO-treated animals were also impaired in long-term retention as seen in their performance in the first trial of the second day of active place avoidance (Figures 2). NAC enhanced MINO action, but had little effect on active place avoidance as a single drug in either massed or spaced training. These data strongly suggest a synergistic effect between MINO plus NAC as seen in the first trial of the second day of active avoidance during massed training as well as throughout spaced training (Figures 2 and 3). Synergy often predicts the most potent drug combinations [7].

MINO plus NAC had little effect on the loss of cortical tissue at the impact site 14 days following moderate CCI. A widespread loss of myelin distal to the impact site was also observed (Figure 4). This widespread myelin loss agrees with previous studies showing widespread axonal damage distal to the impact site following CCI in both rats and mice [30], [31]. Myelin loss was observed in midline white matter structures such as corpus callosum and dorsal hippocampal commissure as well as more lateral white matter structures such as fimbria and internal capsule. The demonstration that myelin loss with MINO plus NAC was no different than MINO alone suggested that NAC acted indifferently to MINO.

Improved white matter function is the likely result of better preservation of myelinated fibers. Injured animals had similar performance in passive place avoidance whether they received saline- or drug-treatment (Table 1). In contrast, MINO-treated animals partially acquired active place avoidance during massed training (Figure 2). Rats perceive both distal and proximal cues in active place avoidance but only the distal cues provide information needed to avoid the shock zone [11]. Thus, avoiding the shock zone likely requires the concerted action of multiple brain regions [13], [32], [33]. Both hippocampi as well as hippocampal LTP are necessary for active place avoidance learning and memory retention [12], [13], [34]. The hippocampal commissure provides monosynaptic connections between the two hippocamppi; myelin loss in the hippocampal commissure was prevented by MINO plus NAC [35]. Myelin loss in the hippocampal commissure in moderately injured animals may result in a conduction block that can impair hippocampal function (Figure 4) [35]. Improved behavior in rats receiving MINO or MINO plus NAC may result from partial prevention of myelin loss. A similar prevention of myelin loss occurred with MINO or MINO plus NAC. These data suggest that the prevention of myelin loss does not fully explain why MINO plus NAC is more potent than MINO alone. MINO plus NAC treatment improved active place avoidance in both the massed and spaced versions of training active avoidance. MINO plus NAC-treated rats improved long-term memory despite a similar prevention of myelin loss a rats receiving MINO alone.

Either MINO or NAC inhibit IL-1β formation when dosed or within 30 minutes after experimental TBI [18], [27]. A potent, synergistic inhibition of IL-1β formation was seen when MINO plus NAC was dosed 3 hours before injury (Figure 5). No change in IL-1β formation, however, occurred when the drugs were dosed one hour post-injury. At identical doses used in this study, IL-1β formation was inhibited when MINO was dosed 30 minutes or NAC 15 minutes post-injury [18], [27]. These data suggest that IL-1β formation that occurs within 30 minutes of experimental TBI can be blocked by MINO or NAC. In contrast, later IL-1β formation remains unchanged after MINO or NAC treatment. These data also suggest that the cognitive and memory improvements produced by MINO plus NAC dosed 1 hour after injury occurs independently of IL-1β formation.

The actual pharmacological targets of MINO are not clear, but the downstream effects of MINO include suppression of inflammation and apoptosis [36], [37]. More is known about the action of NAC. NAC directly elevates glutathione in the brain that scavenges cytoplasmic reactive oxygen species. The anti-oxidant effect of NAC results in widespread changes in both signaling molecules and transcription factors [26]. NAC may also increase extrasynaptic glutamate by increasing the activity of glutamate-cystine transporters [38]. Thus, there are large and complex potential interactions of MINO and NAC. These interactions potentially impact on many aspects of brain function that are more likely to be revealed by assessing complex brain functions using assays such as active place avoidance behavior rather than with tests with more focused outcomes such as inhibition of IL-1 formation (Figures 3 and 5).

MINO had more efficacy than NAC in limiting injury following moderate CCI. MINO improved performance on massed active place avoidance (Figures 1 and 2) and prevented myelin loss (Figure 5). NAC showed little efficacy as a single drug. NAC, however, potently reduces brain redox, suggesting that MINO may be more potent in a more reducing environment [26].

MINO plus NAC are in multiple clinical trials as individual drugs against a variety of neurodegenerative diseases. Although none of these trials have led to the clinical use of these drugs to treat neurodegeneration, the sheer number of ongoing trials attests to both the efficacy and safety of MINO plus NAC as individual drugs. The finding that MINO plus NAC can act synergistically suggests the combination may show enhanced efficacy not only against TBI, but other neurological diseases as well.

## Materials and Methods

### Ethics statement

All animal work must have been conducted according to relevant national and international guidelines. In addition, all procedures were done according to protocol 03-054-10 that has been approved by the SUNY-Downstate Institutional Animal Care and Use Committee (Assurance number A3260-01).

### Production and treatment of CCI

Moderate CCI was produced as described by Abdel Baki et al. [8]. Briefly, Sprague-Dawley rats (250–300g, Charles River, Wilmington, MA) were anesthetized using isoflurane (3–5%) in oxygen (0.8 L/min) and a unilateral craniotomy (6.0 mm) was performed midway between lambda and bregma. A 5.0 mm impactor tip compressed the cortex to a depth of 2.5 mm at 4 m/sec (myNeurolab.com, St. Louis, MO). Rats received three injections of single drugs or drug combinations one hour and one and two days after surgery. The doses of minocycline (45 mg/kg, [18], N-acetylcysteine (150 mg/kg, [21], cyclosporine A, (20mg/kg, [23], progesterone (32 mg/kg, [24], and simvastatin (1mg/kg, [25] were chosen from previous studies that improved an assortment of brain injury parameters. All drugs were injected intraperitoneally, except for simvastatin that was administered by oral gavage.

### Behavioral Analysis

Rats were tested on a hierarchy of computer-controlled behavioral tasks using a commercial hardware and software system (ITS, Bio-Signal Group, Brooklyn, NY) as described by Abdel Baki, et al., 2009 [8]. All tasks were done on an 82-cm diameter metal arena with a 40-cm high transparent wall that allowed the rat to view prominent visual cues in the room. Motor function returns to preinjury levels by 7 days thus ensuring that altered motor function does not alter performance on the hierarchy of behavioral tasks (Table 1). Beginning seven days after surgery, rats were tested on three behavioral tasks. During each task, the rat's location was identified each 33 ms by the video-tracking system, which delivered a mild electrical foot shock according to the parameters of the software algorithm that controlled the experiment (Tracker, Bio-Signal Group, Brooklyn, NY). The data were stored for off-line computation of a variety of behavioral parameters using TrackAnalysis software (Bio-Signal Group, Brooklyn, NY).

The first task of the hierarchy was an open-field test. The arena was stationary and no shocks were administered. The distance traveled assayed habituation to the arena, a measure of innate exploratory behavior. Immediately after open-field testing, rats received the second task of the hierarchy. For this passive place avoidance test, a computer-controlled 60° shock zone was added to the stationary arena. Rats received four 10-minute tests of passive place avoidance with a 10-minute intertrial interval. The total number of shock zone entrances measured the ability to avoid the shock zone and the distance traveled assayed an aversively conditioned ability to inhibit movement. The following day, rats were tested in the active place avoidance task using either the massed or the spaced training protocol. During active place avoidance the arena rotated at 1 rpm. The arena also has a stationary 60° shock zone that remained fixed across trials. During massed training, rats received six 10-minute trials for either one or two days with a 10-minute intertrial interval. The number of shock zone entrances assayed learning that relied on hippocampus-dependent short-term, but not long-term memory [12]. In the case of two days of active place avoidance, the first trial of the second day (the 7^th^ trial of 12 total trials) assayed 24-hour memory retention of the shock zone location. During spaced active placed avoidance training, rats received one 20-minute trial each day for 15 days. Time elapsed before the first entrance into the shock zone assayed 24-hour task retention during learning and the number of entrances assessed task acquisition that is influenced by a variety of factors including motivation to avoid shock, sensitivity to shock, anxiety, stress, short-term memory, perceptual salience of cues, ability to navigate, ability to inhibit locomotion, ability to initiate escape behaviors, choice of behavioral strategy, ability to select appropriate behaviors, cognitive control, attention, in addition to 24-hr retention [39]. Average speed was determined by computing speed every 2 seconds. Linearity assessed path trajectory and is defined as the average of the following ratio:
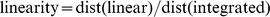
Dist(integrated) is the sum of the distances moved each 33 ms in the 2-second interval, and dist(linear) is the linear distance between the locations at the start and end of the 2-second interval. Behavioral parameters were analyzed using 2-way repeated-measures ANOVA by comparing treatment of groups across trials. A primary goal was to measure asymptotic behavior differences between groups, therefore, 1-way ANOVA planned comparisons evaluated group differences on a particular training day or on performance across an entire training protocol. When appropriate, Newman-Keuls post-hoc tests compared group differences.

### Histological Analysis

Fourteen days after sham- or moderate-CCI, urethane (1.2 g/kg, i.p.) followed by transcardial infusion with paraformaldehyde (4% (w/v)), coronal brain sections were prepared as described in Abdel Baki, et al., 2009 [8]. Coronal sections were stained using Luxol fast blue according to the manufacturer's instructions (American Mastertech, Lodi, CA). Digital images were prepared and the amount of Luxol Fast Blue staining was assayed using Image J software in regions of interests (ROIs) within the corpus callosum, dorsal hippocampal commissure, fimbria, internal capsule, fornix, and mamallothalamic tract in coronal sections located −3.10–3.45 mm from Bregma [40]. To determine the amount of Luxol fast blue staining that was due to myelin staining, staining intensity in the ROIs was subtracted from staining intensity values from the stratum radiatum of the hippocampus that contained the umyelinated Schaffer collateral. A recent study showed a positive correlation between luxol fast blue staining and the parameters of fractional anisotropy and radial diffusivity in rats following experimental TBI [41].

Lesion volume in the ipsilateral cortex was determined by the method of Raghupathi and Huh, 2007 [42]. Lesion volume (expressed as the percentage difference from the contralateral cortex) was calculated from a series of adjacent coronal sections using the formula:
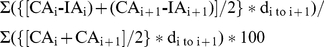
where d_i to i+1_ is the distance between sections i and i+1; IA_i_ and IA_i+1_ are the areas of lesioned cortex; and CA_i_ and CA_i+1_ are the corresponding areas of unlesioned cortex. Lesion volume in all groups was normalized to values from CCI-saline rats.

### Assay of IL-1β production

To examine the effect of dosing drugs before injury, rats were treated with saline MINO, NAC or MINO plus NAC three hours before sham-CCI or moderate CCI. One hour after surgery, rats were anesthetized with halothane and decaptitated. The hippocampi were rapidly removed and frozen in liquid nitrogen. To examine an effect of dosing drugs after injury, rats received sham- or moderate CCI and were treated with saline or drugs one hour after injury. Three hours after saline or drug treatment, hippocamppi were removed in an identical manner as the preinjury study.

Protein was extracted by grinding the tissue while rapidly thawing in Tris-HCl, (120mM, pH 6.8) and SDS (4%, w/v) at 100°C for 10 minutes followed by centrifugation at 13,000g for 5 minutes. Protein extract (30ug) was analyzed by immunoblot using an antibody against β-tubulin (1/1000 dilution; ab7792, Abcam, Inc., Cambridge, MA) and against IL-1β (1/1000 dilution, #2021, Cell Signaling, Inc., Danvers, MA). Group comparisons were made by ANOVA followed by Newman-Keuls post-hoc tests. Significance was p<0.05. All values are reported as the mean ± SEM.
